# Meta-analysis of the global distribution of clinically relevant *CYP2C8* alleles and their inferred functional consequences

**DOI:** 10.1186/s40246-024-00610-y

**Published:** 2024-04-22

**Authors:** Mahamadou D. Camara, Yitian Zhou, Taís Nóbrega De Sousa, José P. Gil, Abdoulaye A. Djimde, Volker M. Lauschke

**Affiliations:** 1 Department of Physiology and Pharmacology, Karolinska Institutet, 171 77 Stockholm, Sweden; 2 Malaria Research and Training Center, Department of Epidemiology of Parasitic Diseases, Faculty of Pharmacy, University of Science, Techniques and Technologies, Bamako, Mali; 3 Department of Microbiology and Tumor Cell Biology, Karolinska Institutet, Stockholm, Sweden; 4 Molecular Biology and Malaria Immunology Research Group, Instituto René Rachou, Fundação Oswaldo Cruz (FIOCRUZ), Belo Horizonte, Brazil; 5 Institute of Hygiene and Tropical Medicine, Global Health and Tropical Medicine, Nova University of Lisbon, Lisbon, Portugal; 6 Dr Margarete Fischer-Bosch Institute of Clinical Pharmacology, Stuttgart, Germany; 7 University of Tübingen, Tübingen, Germany

**Keywords:** Allele frequency, Amodiaquine, Drug metabolism, Pharmacogenetics, Precision medicine, Seasonal malaria chemoprevention

## Abstract

**Background:**

CYP2C8 is responsible for the metabolism of 5% of clinically prescribed drugs, including antimalarials, anti-cancer and anti-inflammatory drugs. Genetic variability is an important factor that influences CYP2C8 activity and modulates the pharmacokinetics, efficacy and safety of its substrates.

**Results:**

We profiled the genetic landscape of *CYP2C8* variability using data from 96 original studies and data repositories that included a total of 33,185 unrelated participants across 44 countries and 43 ethnic groups. The reduced function allele CYP2C8*2 was most common in West and Central Africa with frequencies of 16–36.9%, whereas it was rare in Europe and Asia (< 2%). In contrast, CYP2C8*3 and CYP2C8*4 were common throughout Europe and the Americas (6.9–19.8% for *3 and 2.3–7.5% for *4), but rare in African and East Asian populations. Importantly, we observe pronounced differences (> 2.3-fold) between neighboring countries and even between geographically overlapping populations. Overall, we found that 20–60% of individuals in Africa and Europe carry at least one *CYP2C8* allele associated with reduced metabolism and increased adverse event risk of the anti-malarial amodiaquine. Furthermore, up to 60% of individuals of West African ancestry harbored variants that reduced the clearance of pioglitazone, repaglinide, paclitaxel and ibuprofen. In contrast, reduced function alleles are only found in < 2% of East Asian and 8.3–12.8% of South and West Asian individuals.

**Conclusions:**

Combined, the presented analyses mapped the genetic and inferred functional variability of *CYP2C8* with high ethnogeographic resolution. These results can serve as a valuable resource for *CYP2C8* allele frequencies and distribution estimates of CYP2C8 phenotypes that could help identify populations at risk upon treatment with CYP2C8 substrates. The high variability between ethnic groups incentivizes high-resolution pharmacogenetic profiling to guide precision medicine and maximize its socioeconomic benefits, particularly for understudied populations with distinct genetic profiles.

**Supplementary Information:**

The online version contains supplementary material available at 10.1186/s40246-024-00610-y.

## Introduction

The hepatic cytochrome P450 enzyme CYP2C8 is responsible for the metabolism of multiple endogenous compounds and clinically relevant xenobiotics, including antimalarials, nonsteroidal anti-inflammatory drugs, thiazolidinediones, meglitinides, as well as taxanes [1]. The gene encoding CYP2C8 is highly polymorphic and, by now, more than 700 variants in *CYP2C8* have been identified [2]. Genetic variability in *CYP2C8* can be an important source of interindividual variability in pharmacological response and toxicity. For instance, variants associated with altered CYP2C8 activity decreased clearance and increased neurotoxicity of paclitaxel [3]. Similarly, reduced CYP2C8 activity increased the number of adverse drug reactions (ADRs) of amodiaquine by almost 60% [4, 5].

Among all *CYP2C8* variations, the CYP2C8*2 and CYP2C8*3 haplotypes are most frequent. CYP2C8*2 is defined by an p.I269F amino acid substitution while the CYP2C8*3 allele comprises two variants encoding the missense variants p.R139K and p.K399R, respectively. The enzymes encoded by CYP2C8*2 and CYP2C8*3 exhibit overall reduced metabolism of CYP2C8 substrates in most but not all in vitro studies [6–9] and their distribution is known to differ across populations and ethnicities. CYP2C8*2 is more prevalent in African populations with minor allele frequencies (MAFs) pivoting around 15%, whereas CYP2C8*3 is more frequent in Caucasians with MAFs between 7.5 and 14.3% [10–13]. In addition to these common alleles, there are a multitude of rare *CYP2C8* alleles with global minor allele frequencies < 1% that affect gene function, including CYP2C8*4, *5, *7 and *14.

Importantly, however, current studies mapping *CYP2C8* variability either evaluated large aggregated superpopulations or analyzed frequencies in one or few specific populations or countries. To provide a global overview of *CYP2C8* variability with high ethnogeographic resolution, we here integrated variability data from 96 original articles including a total of 33,185 unrelated participants from 44 countries and 43 diverse ethnic groups. We found that haplotype frequencies varied drastically between superpopulations and could even differ > threefold between different geographically overlapping ethnic groups. By aggregating the available genetic variability data into spectra of functional consequences, we infer worldwide patterns of CYP2C8 metabolism and predict the proportion of individuals at risk of responding adversely to CYP2C8 substrates. Combined, these data provide a comprehensive chart of *CYP2C8* variability and its functional effects at the global scale.

## Methods

### Data sources

We performed a systematic literature search in Medline including all original articles that reported *CYP2C8* allele frequencies published before February 2024. Only studies with more than 30 participants were included. In addition, we included data from Finnish and Amish populations from gnomAD [14]. This approach resulted in the identification of 96 studies covering 44 countries and a total of 33,185 individuals (Additional file 2: Table S1). If frequencies for a country or ethnogeographic group were reported by multiple studies, the individual frequencies were aggregated by weighting with the respective cohort sizes.

### Genotype to phenotype translation

There are currently no guidelines by the Clinical Pharmacogenetics Implementation Consortium (CPIC) regarding *CYP2C8* alleles. Consequently, we assigned *CYP2C8* allele function based on the latest PharmGKB allele summary [15]. CYP2C8*3 (rs11572080 and rs10509681; NM_000770.3:c.416G>A and NM_000770.3:c.1196A>G) was considered as a reduced function allele for the metabolism of amodiaquine, whereas it was considered as a normal function allele for other substrates. CYP2C8*2 (rs11572103; NM_000770.3:c.805A>T), *4 (rs1058930; NM_000770.3:c.792C>G), *5 (rs72558196; NM_000770.3:c.475del), *7 (rs72558195; NM_000770.3:c.556C>T), *8 (rs72558195; NM_000770.3:c.556C>G), *11 (rs78637571; NM_000770.3:c.820G>T), *12 (rs3832694; NM_000770.3:c.1382_1384del) and *14 (rs188934928; NM_000770.3:c.712G>C) were considered as decreased activity alleles for all substrates. Diplotypes were calculated on the basis of the Hardy–Weinberg equation. Individuals without a reduced function allele were considered as normal metabolizers (NMs), while individuals carrying one or two decreased function alleles were as intermediate metabolizers (IMs) and poor metabolizers (PMs), respectively. Frequencies of the reference allele (CYP2C8*1) were calculated as f_1_ = 1 − Σ_i_ f_i_, with f_i_ being the frequency of each analyzed variants allele *i* for which frequency information was available.

## Results

### Geographic distribution of functionally important CYP2C8 alleles

**Fig. 1:**
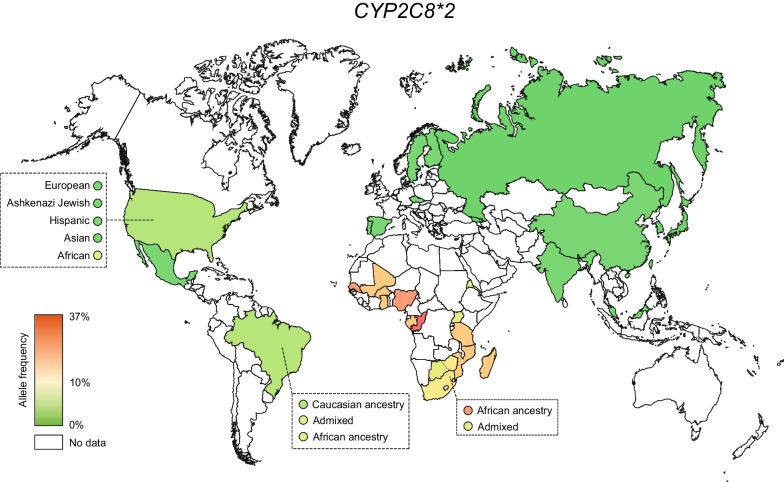
Global distribution of CYP2C8*2. National allele frequencies are shown across 30 countries with the highest frequency in red, the average frequency across all countries in yellow, and the lowest frequency in green. Countries without available *CYP2C8* frequency information are shown in white

To understand the global distribution of *CYP2C8* variability, we first integrated allele frequency data of the three most common functionally relevant alleles, CYP2C8*2, CYP2C8*3 and CYP2C8*4. CYP2C8*2 was very common throughout Africa with frequencies ranging between 6% in Eritrea to 36.9% in Congo (Fig. 1, Table 1). Overall, there was a tendency towards higher frequencies in Western and Central Sub-Saharan Africa (16–36.9%) compared to Southern (11.1–16.2%) and Eastern Africa (5.9–17.3%). In Europe, the CYP2C8*2 allele is mostly rare with highest frequencies in Portugal (1.2%) and Spain (1.6%). In Asia, the allele is mostly undetectable with the exception of some areas in South and Southeast Asia, such as India (1.8%) and Malaysia (1.9%).

**Table 1:** Global frequencies of the most common *CYP2C8* alleles

Country	N (participants)	2 (%)	3 (%)	4 (%)
*Africa*				
Tanzania	1089	17.3	2.3	0.6
Uganda	523	10.4	0	–
Madagascar	153	15	–	–
Eritrea	376	6	4.7	–
Nigeria	220	19.1	0	–
Mali	206	16	0	0
Burkina-Faso	889	18	0.4	0
Senegal	88	22.2	–	–
Ghana	589	18	0	0
Botswana	544	11.1	–	–
Mozambique	424	16.2	4.9	0.6
South Africa and Zimbabwe	490	14.5	–	1
Gabon	48	16.7	–	–
Congo	213	36.9	–	–
*Americas*				
Mexico	241	0.9	7.5	4.6
USA	2878	2.4	7.5	2.3
Ecuador	579	–	8.1	–
Brazil	1403	5.3	8.6	3.5
Bolivia	129	–	3.9	–
*Europe*				
Denmark	482	–	9.9	–
Norway	309	–	9.1	–
Sweden	2496	0.6	9.8	5.6
Faroese Island	312	–	6.9	–
Finland	5847	0.003	11.5	5.6
UK	219	0.4	10.8	7.5
Scotland	719	–	15.1	–
Italy	33	–	10.6	–
Spain	933	1.6	15.8	5.1
Portugal	164	1.2	19.8	6.4
Russia	479	0	8.7	–
Czech Republic	161	0.3	10.9	5.9
Bulgaria	736	–	11.2	–
Hungary	826	–	8.8	3.9
Germany	2827	–	10.1	–
Netherlands	96	–	9.9	4.6
*Asia*				
Malaysia	258	1.9	1.9	0
Cambodia	75	–	0	–
China	1287	0.2	2	1
Japan	1102	0	0	0
Korea	146	0	0	0
India	985	1.8	3	2.4
Jordan	381	–	4.3	6.6
*Oceania*				
Australia	210	–	8.8	3.8

For references, we refer the interested reader to Additional file 2: Table S1

**Fig. 2:**
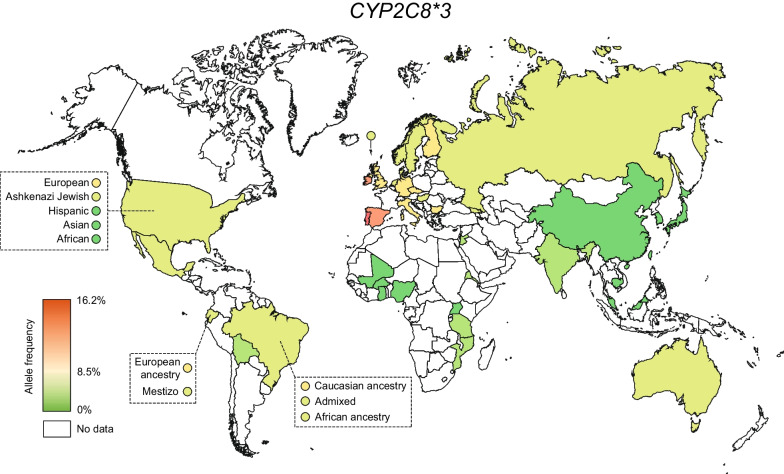
Global distribution of CYP2C8*3. National allele frequencies are shown across 36 countries with the highest frequency in red, the average frequency across all countries in yellow, and the lowest frequency in green. Countries without available *CYP2C8* allele frequency information are shown in white

**Fig. 3:**
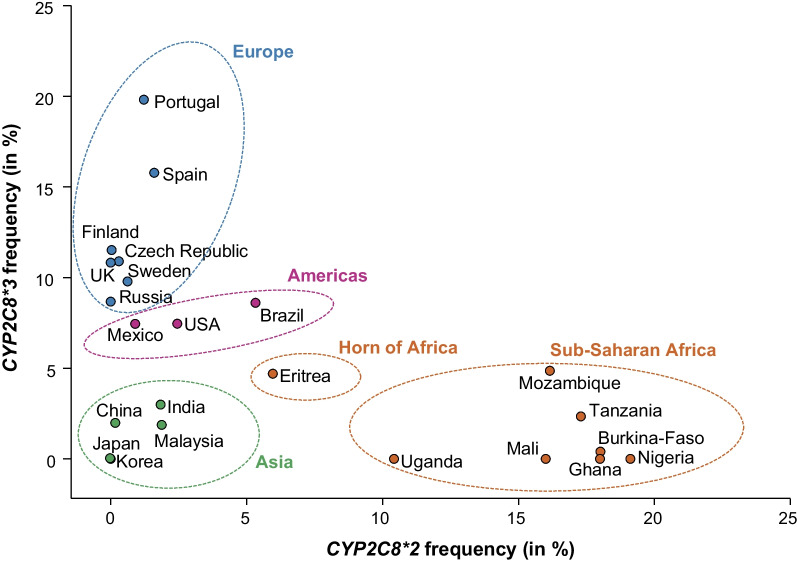
The frequencies of CYP2C8*2 and CYP2C8*3 are in anti-correlation. The association between national frequencies of CYP2C8*2 and CYP2C8*3 are shown for 22 countries. Note that allele frequency patterns cluster by geographical region and are in significant anti-correlation (r = 0.49; *p* = 0.0099)

While CYP2C8*2 was most common in Africa, CYP2C8*3 is mostly rare in Africa (0–4.9%) and most common in Europe and throughout the Americas (Fig. 2, Table 1). The highest frequencies were observed on the Iberian Peninsula (15.8–19.8%) and Scotland (15.1%) while lowest frequencies were found on the Faroese Islands (6.9%), and in Hungary (8.8%). In Asia, the CYP2C8*3 allele was similarly rare as CYP2C8*2 with a few exceptions, such as relatively high frequencies in Jordan (4.3%) and India (3%). Population-specific differences in allele frequency could also be identified in self-reported data from multi-ethnic countries, such as the US and Brazil, where CYP2C8*3 was highly prevalent in individuals of European heritage but much less common in African and Asian groups. Inversely, CYP2C8*2 was common in participants of African ancestry, but rare in Europeans or Ashkenazi Jews. Combined, these data revealed that CYP2C8*2 and CYP2C8*3 are mostly in anticorrelation with the exception of Asia where both alleles were mostly rare (Fig. 3).

**Fig. 4:**
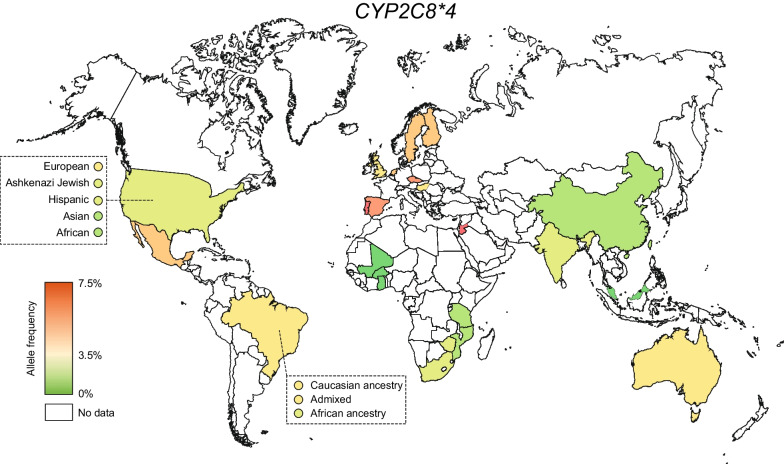
Global distribution of CYP2C8*4. National allele frequencies are shown across 25 countries with the highest frequency in red, the average frequency across all countries in yellow, and the lowest frequency in green. Countries without available *CYP2C8* frequency information are shown in white

Distributions of CYP2C8*4 were overall similar to that of CYP2C8*3 (Table 1, Fig. 4). This allele was common in many European countries (up to 7.5% in the United Kingdom), but rare or absent throughout Africa and most of Asia. Notably, CYP2C8*4 was the most common *CYP2C8* allele in Jordan, suggesting that this haplotype might have a relatively higher relevance in the Middle East. Other *CYP2C8* alleles whose frequencies have been reported include CYP2C8*5, CYP2C8*6, CYP2C8*7, CYP2C8*8, CYP2C8*9, CYP2C8*10, CYP2C8*11, CYP2C8*12, CYP2C8*13, and CYP2C8*14. All of these alleles are rare, and their prevalence has only been investigated in one or few geographical regions, mostly in Asia (Additional file 3: Table S2).

### CYP2C8 variability across ethnic groups

**Fig. 5:**
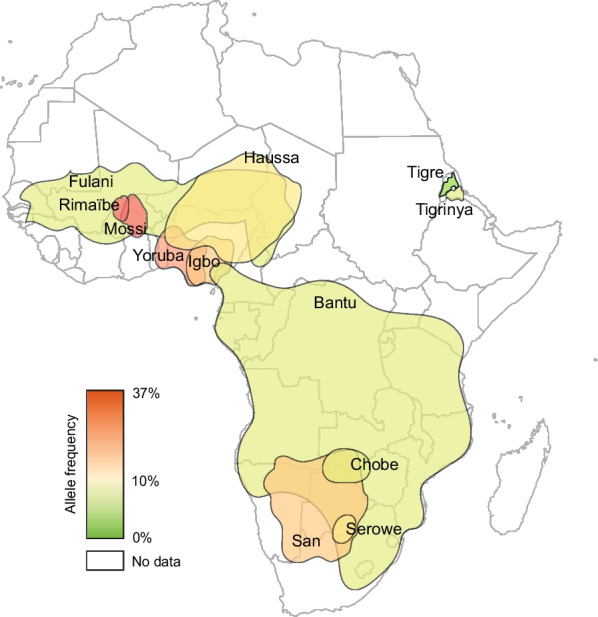
CYP2C8*2 distribution strongly differs between ethnogeographic groups in Sub-Saharan Africa. The expansion and population frequencies of the indicated groups are shown. Color-code indicates the highest frequency in red, the average frequency across all shown groups in yellow, and the lowest frequency in green

Next, we focused our analyses on allele frequency distributions across ethnic groups. Overall, we extracted information from 43 ethnic groups across Africa, Asia, Europe and the Americas (Table 2). CYP2C8*2 was common across African populations (4.8–23.4%) as well as in admixed populations in the Americas (4–6.3%), whereas it was rare or absent in individuals of European or Asian Americans or individuals of Norse, Gaelic, or Han ancestry. The overall highest frequencies were recorded in the Mossi (23.4%) and Rimaibe (23%) from Burkina-Faso in Western Africa, whereas frequencies in the Fulani population were considerably lower (9.9%) despite its geographical overlap (Fig. 5). These results demonstrated that differences between populations are more pronounced when ancestry or ethnicity is used for stratification rather than geographic factors. This highlights the importance of depicting genetic variability with high ethnogeographic resolution.

**Table 2:** *CYP2C8* allele frequencies across different ethnogeographic groups

Ethnic group	N (participants)	*2 (%)	*3 (%)	*4 (%)
*Africa*				
Hausa	23	15.2	0	–
Igbo	43	17.4	0	–
Yoruba	100	21.5	0	–
Fulani	71	9.9	–	–
Mossi	248	23.4	–	–
Rimaïbe	187	23	–	–
Tigre	62	4.8	1.6	–
Tigrinya	250	6.8	5	–
Bantu	384	8.5	–	–
Chobe (Bantu)	157	7.3	–	–
Serowe/Palapye (Bantu)	227	10.2	–	–
Ghanzi (San)	160	17.5	–	–
*Americas*				
Caucasian/European American	824	0.1	11.3	3.6
African American	534	8.2	1.9	1
Asian American	345	0.3	0.2	0.9
Han Chinese/Asian	48	0	0	0
Japanese American	48	0	0	0
Hispanic American	248	2.2	7.6	1.8
Ashkenazi Jewish	249	0.4	7.6	2
Amish	456	0	15.2	5
Spaniard	282	–	10.3	–
Ecuadorian Mestizo	297	–	6.1	–
Brazilian (Caucasian ancestry)	342	3.6	12.1	3.6
Brazilian (Admixed)	350	6.3	4.9	3.4
Brazilian (African ancestry)	342	9.7	5.1	2.2
*Europe*				
Mixed Norse and Gaelic origins	312	–	6.9	–
Avars	100	–	5.5	–
Dargins	100	–	10	–
Laks	100	–	6.5	–
Kumyks	100	–	8.5	–
Chechens	88	–	–	12.2
Roma	397	–	–	2.1
Hungarian	412	2.1	8.8	5.8
*Asia*				
Western Chinese	136	–	4.4	–
Han	380	0	0	0
Uighur	153	0.3	2.9	2.3
Hui	158	0	1.6	0.6
Mongolian in China	127	0.4	1.6	1.2
Tamilians	608	1.6	3	–
North Indian	254	2.8	3.9	3.6
Circassians	93	–	0	1.7
Jordanian-Arabs	200	–	8.2	6.5
*Oceania*				
Tiwi Indians	492	0	0.1	0

For references, we refer the interested reader to Additional file 2: Table S1

The highest frequencies of CYP2C8*3 were found in the Amish (15.2%) and Americans of European ancestry (12.1–10.3%), followed by Dargins (10%) and Kumyks (8.5%) from the Caucasus and Jordanian Arabs (8.2%). Very similar results were obtained for CYP2C8*4, which was also most prevalent in the Caucasus and Western Asia (12.2% in Chechens and 6.5% in Jordanian Arabs). Taken together, these results corroborate that CYP2C8*3 and CYP2C8*4 are common throughout most of Europe and its interface with Western Asia. In Africa, CYP2C8*3 was only found in the Tigre (1.6%) and Tigrinya peoples (5%) in the East of the continent, whereas no data were reported for CYP2C8*4 in African ethnic groups.

### Translation of genetic variability into CYP2C8 metabolizer phenotypes

To understand the functional impact of the observed genetic differences, we used the available data to estimate CYP2C8 metabolizer phenotypes. Notably, there are currently no generally accepted assignments of activity scores to the individual variant alleles. CYP2C8*2 has been repeatedly associated with the reduced clearance of CYP2C8 substrates, likely due to a destabilization of the gene product, and we thus considered this allele as decreased function studies [6–8]. We also considered CYP2C8*4, CYP2C8*5, CYP2C8*7, CYP2C8*8, CYP2C8*11, CYP2C8*12 and CYP2C8*14 as decreased function alleles [16–20]. In contrast to the aforementioned haplotypes, the functional consequences of CYP2C8*3 are substrate-specific. CYP2C8*3 does not decrease metabolism of paclitaxel, pioglitazone, repaglinide, cinitapride, or ibuprofen [8, 21–23], whereas the available evidence points to a reduction of its catalytic activity for the metabolism of amodiaquine to desethylamodiaquine (DEAQ) [4, 5]. Previous studies suggested that the mechanism underlying the reduced activity of CYP2C8.3 might be the slower electron transfer from POR, which results in a slower catalytic cycle [24, 25]. To account for this substrate-specificity of CYP2C8*3, we inferred separate activity profile for CYP2C8 substrates where CYP2C8*3 was considered as a normal activity allele, such as pioglitazone, repaglinide, paclitaxel and ibuprofen, and for amodiaquine, where CYP2C8*3 was associated with decreased function.

**Fig. 6:**
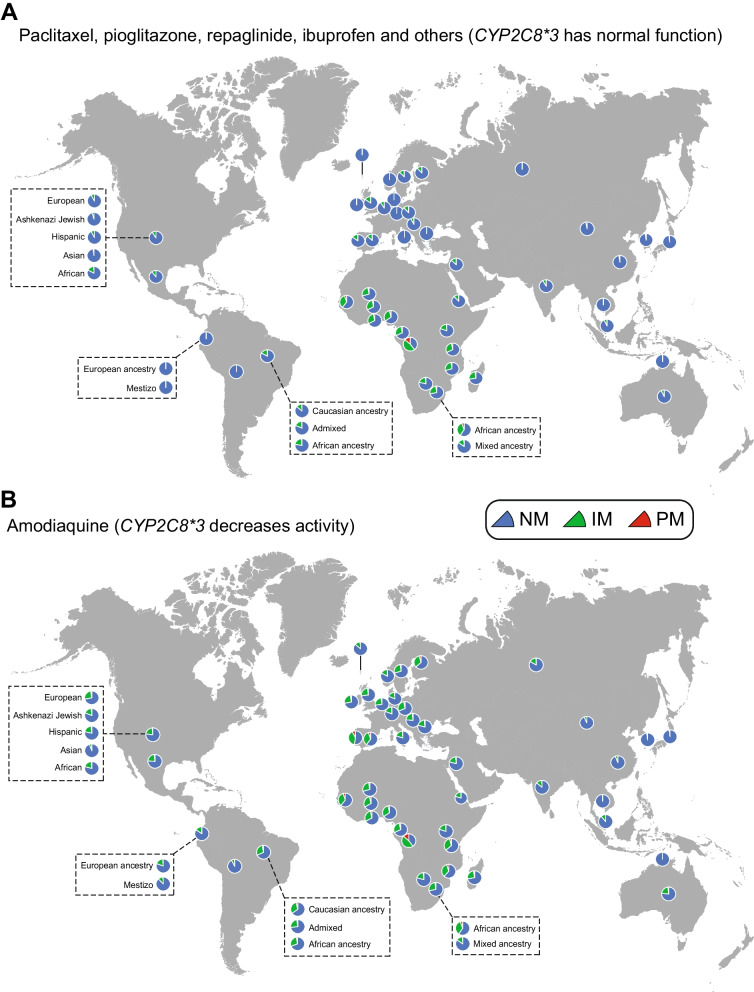
Map of inferred CYP2C8 metabolizer phenotypes Pie charts show the fraction of normal metabolizer (NM, in blue), intermediate metabolizer (IM, in green) and poor metabolizer (NM, in red) for representative countries. IMs and PMs were defined as individuals carrying one or two reduced function alleles, respectively.** A**, CYP2C8*3 is considered as a normal function allele. **B**, CYP2C8*3 is considered as a reduced function allele

For substrates where CYP2C8*3 was considered as an allele with normal function, CYP2C8 IMs and PMs were largely limited to Africa, diasporas of individuals with African ancestry as well as admixed populations (Fig. 6A; Additional file 1: Fig. S1A; Table 3). The fraction of intermediate metabolizers pivots around 20%-30% but can exceed 45% in specific groups in West Sub-Saharan Africa. Furthermore, up to 13.6% of individuals of African ancestry are homozygous or compound heterozygous for *CYP2C8* reduced function alleles, suggesting that increased vigilance is advised upon prescription of CYP2C8 substrates to avoid overexposure, particularly in these populations. In contrast, maximally 15% and 0.6% were classified as IMs and PMs in Europe and Asia with highest prevalence on the Iberian Peninsula and in the Middle East.

**Table 3:** Frequencies of inferred CYP2C8 metabolizer phenotype

Country	NMs (%)	IMs (%)	PMs (%)
*Africa*			
Tanzania	63.6/67.4	32.3/29.4	4.1/3.2
Uganda	80.2	18.7	1.1
Madagascar	72.2	25.5	2.3
Eritrea	79.8/88.4	19.1/11.2	1.1/0.4
Nigeria	65.4	30.9	3.7
Mali	70.5	26.9	2.6
Burkina-Faso	66.6/67.2	30/29.5	3.4/3.3
Senegal	60.6	34.5	4.9
Ghana	67.3	29.5	3.2
Botswana	79	19.8	1.2
Mozambique	61.5/69.4	33.8/27.8	4.7/2.8
South Africa and Zimbabwe	71.4	26.2	2.4
Gabon	67.8	29.1	3.1
Congo	39.9	46.5	13.6
*Americas*			
Mexico	75.8/89.4	22.5/10.3	1.7/0.3
USA	77.1/90.7	21.4/9.1	1.5/0.2
Ecuador	84.4/100	14.9/0	0.7/0
Brazil	68.2/83.1	28.8/16.1	3/0.8
Bolivia	92.4/100	7.5/0	0.1/0
*Europe*			
Denmark	81.2/100	17.8/0	1/0
Norway	82.7/100	16.5/0	0.8/0
Sweden	70.6/88	26.8/11.6	2.6/0.4
Faroese Island	86.7/100	12.8/0	0.5/0
Finland	69.6/88.5	27.7/11.1	2.7/0.4
UK	72.8/84.6	25/14.8	2.2/0.6
Scotland	72.1/100	25.6/0	2.3/0
Italy	79.9/100	19/0	1.1/0
Spain	60.2/87.1	34.8/12.4	5/0.5
Portugal	52.7/85.3	39.8/14.1	7.5/0.6
Russia	83.4/100	15.8/0	0.8/0
Czech Republic	68.7/88	28.4/11.6	2.9/0.4
Bulgaria	78.8/100	19.9/0	1.3/0
Hungary	76.1/92.3	22.3/7.6	1.6/0.1
Germany	80.9/100	18.1/0	1/0
Netherlands	73/90.9	24.9/8.9	2.1/0.2
*Asia*			
Malaysia	89/92.6	10.7/7.3	0.3/0.1
Cambodia	100	0	0
China	93.9/97.7	6/2.3	0.1/0
Japan	98.4	1.6	< 0.1
Korea	98	2	< 0.1
India	86/91.7	13.5/8.1	0.5/0.2
Jordan	79.3/87.2	19.5/12.4	1.2/0.4
*Oceania*			
Australia	76.4/92.5	22/7.3	1.6/0.2

The percentages of the different metabolizer phenotypes are shown for amodiaquine (before the “/”), where CYP2C8*3 is considered a reduced function allele, and for other substrates (after the “/”), where CYP2C8*3 is associated with normal function

*NM* Normal metabolizers; *IM* Intermediate metabolizers; *PM* Poor metabolizers

For substrates for which CYP2C8*3 was associated with reduced metabolic clearance, the distribution of IMs and PMs expanded from Africa to also include European populations (Fig. 6B; Additional file 1: Fig. S1B; Table 3). Prevalence of individuals with predicted reduced metabolic CYP2C8 activity increased particularly in Portugal (39.8% IMs, 7.5% PMs), Spain (34.8% IMs, 5% PMs) and Brazil (28.8% IMs, 3% PMs), but was generally high throughout Europe and Western Asia. In contrast, the fraction of individuals with reduced CYP2C8 metabolism remained low in Southeast Asia, irrespective of whether CYP2C8*3 was considered a normal or reduced function allele.

## Discussion

*CYP2C8* variability can have a major impact on the pharmacokinetics, response and toxicity of a wide range of drugs including the antimalarial amodiaquine, the antiarrythmic amiodarone, the antiemetic cinitapride, various antidiabetics as well as the taxane paclitaxel [26]. Reduced activity of CYP2C8 is predominantly caused by CYP2C8*2, CYP2C8*3 and CYP2C8*4. CYP2C8*2 constitutes the predominant *CYP2C8* variant allele in African populations, whereas the allele was rare to absent throughout Asia, Europe and the Americas. Within Africa, we observed striking differences in frequencies between Sub-Saharan West and Central Africa CYP2C8*2 frequencies reached up to 36.9%, whereas frequencies in Eritrea (6%) and East African populations, such as the Tigre (4.8%) and Tigrinya (6.8%) were substantially lower. As such, these data refine the conclusions from previous reviews, which reported African population frequencies between 13 and 20% [10].

Notably, even neighboring countries can have pronounced differences in allele frequencies, which could be explained by differences in population structure between geographically overlapping ethnic groups. For instance, frequencies of CYP2C8*2 differed considerably between Fulani and other sympatric West African groups. The Fulani are a nomadic people mainly living in the Sahel. While historical records suggest that the Fulani originated in Northeast Africa, recent genomic analyses suggest that they have a predominant West African genetic background with clear evidence of two admixture events, the first with a Northeast African population approximately 1800 years ago and the second with a Southwestern European group approximately 300 years ago [27]. CYP2C8*2 prevalence in the Fulani was considerably lower than in Rimaibe, Mossi or Yoruba (9.9% compared to > 21%) but was similar to frequencies found in East African populations (Tigrinya; 6.8%). Interestingly, the Fulani are also known to be less susceptible to malaria infection than other West African groups, such as Mossi or Dogon [28–30]. This underlines the need to conduct further higher resolution studies into the pharmacogenetic diversity of African populations for a more precise application of stratified treatment policies.

Overall, the CYP2C8*3 allele was predominantly found in Europe. In Portugal and Spain, the minor allele frequency was 19.8% and 15.8%, respectively, which is considerably higher than the aggregated population average reported for 589,000 Non-Finnish Europeans reported in gnomAD (11.8%). We observed a slight gradient of CYP2C8*3 frequencies from the European Atlantic coast towards Eastern Europe. Given that the CYP2C8*3-CYP2C9*2 haplotype is assumed to be inherited from the Neandertals [31], its current distribution might be a reflection of the population admixture with modern humans.

CYP2C8 is the enzyme catalyzing oxidation of amodiaquine to DEAQ. Amodiaquine is used both, for malaria treatment and for malaria prevention strategies such as seasonal malaria chemoprevention (SMC) during which children under five years old are administrated amodiaquine monthly for 4 to 5 months per year. Thus, given that 95% of the global incidence of malaria is in Africa, African populations are the main users of amodiaquine. The reduced activity alleles CYP2C8*2 and CYP2C8*3 have been linked to an increased number of adverse drug reactions upon amodiaquine treatment either during prolonged monotherapy or in combination with artemisinin and its derivatives [32, 33]. Mechanistically, it is assumed that amodiaquine toxicity is impacted by the balance between amodiaquine oxidation and the reduction of its quinoneimine metabolite, which can directly bind to cellular components [34, 35]. There is currently, no guideline for the adaptation of treatment regimens in patients with *CYP2C8* decreased activity alleles. Our analysis showed that 20–60% of Africans carry at least one allele associated with lower amodiaquine clearance, which might increase the risk of overexposure and adverse drug reactions (ADRs), particularly in the very populations where the need for this drug is the highest. This could explain the relatively low tolerability of amodiaquine containing combinations compared to other artemisinin combination therapies, with 43% of ASAQ users reporting ADRs across eight randomized controlled clinical trials across nine countries in Sub-Saharan Africa [36].

Given the high prevalence and considerable ethnogeographic variability of reduced activity alleles of *CYP2C8*, it is important to consider population-specific genetic features to optimize local treatment protocols. To be successful however, implementation of precision medicine guidelines requires higher resolution data and more evidence regarding the impact of population-specific variations on treatment outcomes, which might be further emphasized in the context of polypharmacy. This study provides with consolidated maps that integrate that available information while also showcasing where knowledge gaps remain.

## Conclusions

In conclusion, the result of our analysis depicts the global variability of *CYP2C8* alleles and its inferred metabolic consequences. Reduced activity alleles are less frequent in Asia, whereas they are overall common in Africa and Europe affecting around 30–60% of the general population. However, notable differences exist between countries and even between geographically overlapping populations. Consequently, the *CYP2C8* variability profile of the ethnogeographic group in question should be taken into consideration as accurately as possible when planning for treatment with CYP2C8 substrates such as amodiaquine, pioglitazone, repaglinide, paclitaxel and ibuprofen. These results can support the field by highlighting geographical regions and populations where genetic frequency information is currently sparse for more in-depth genetic profiling. Furthermore, we hope that this consolidated and integrated analyses of *CYP2C8* allele and phenotype frequencies will provide a useful resource that could inform policy makers and guide stratified medicine strategies at the global scale.

### Supplementary Information

**Additional file 1: Fig. S1.** Frequencies of intermediate and poor CYP2C8 metabolizers across analyzed countries. Countries are shown in a ranked order from high to low relative abundance of individuals with reduced CYP2C8 metabolism. IMs and PMs were defined as individuals carrying one or two reduced function alleles, respectively. **A** CYP2C8*3 is considered as a normal function allele. **B** CYP2C8*3 is considered as a decreased function allele.

**Additional file 2: Table S1.** References reporting frequencies from different countries and ethnicities.

**Additional file 3: Table S2.** Country-specific frequencies of rare CYP2C8 variants.
