# Supplementary material for: Meta-analysis of the global distribution of clinically relevant CYP2C8 alleles and their inferred functional consequences
Source: Hum Genomics. 2024 Apr 22;18:40. doi: 10.1186/s40246-024-00610-y (PMC11034136; doi:10.1186/s40246-024-00610-y)

Supplementary Figure 1

A

Paclitaxel, pioglitazone, repaglinide, ibuprofen and others (*CYP2C8*3* has normal function)

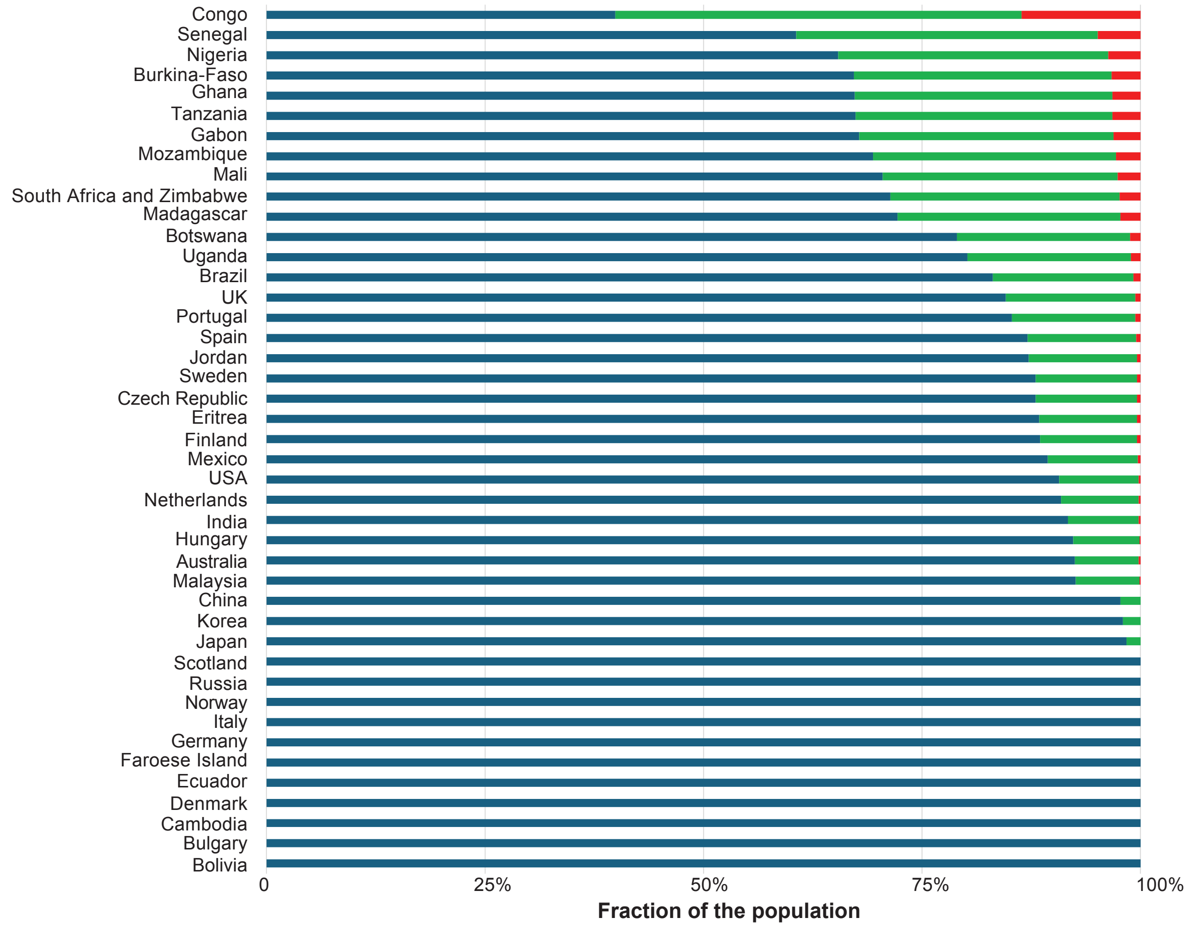

B

Amodiaquine (*CYP2C8*3* decreases activity)

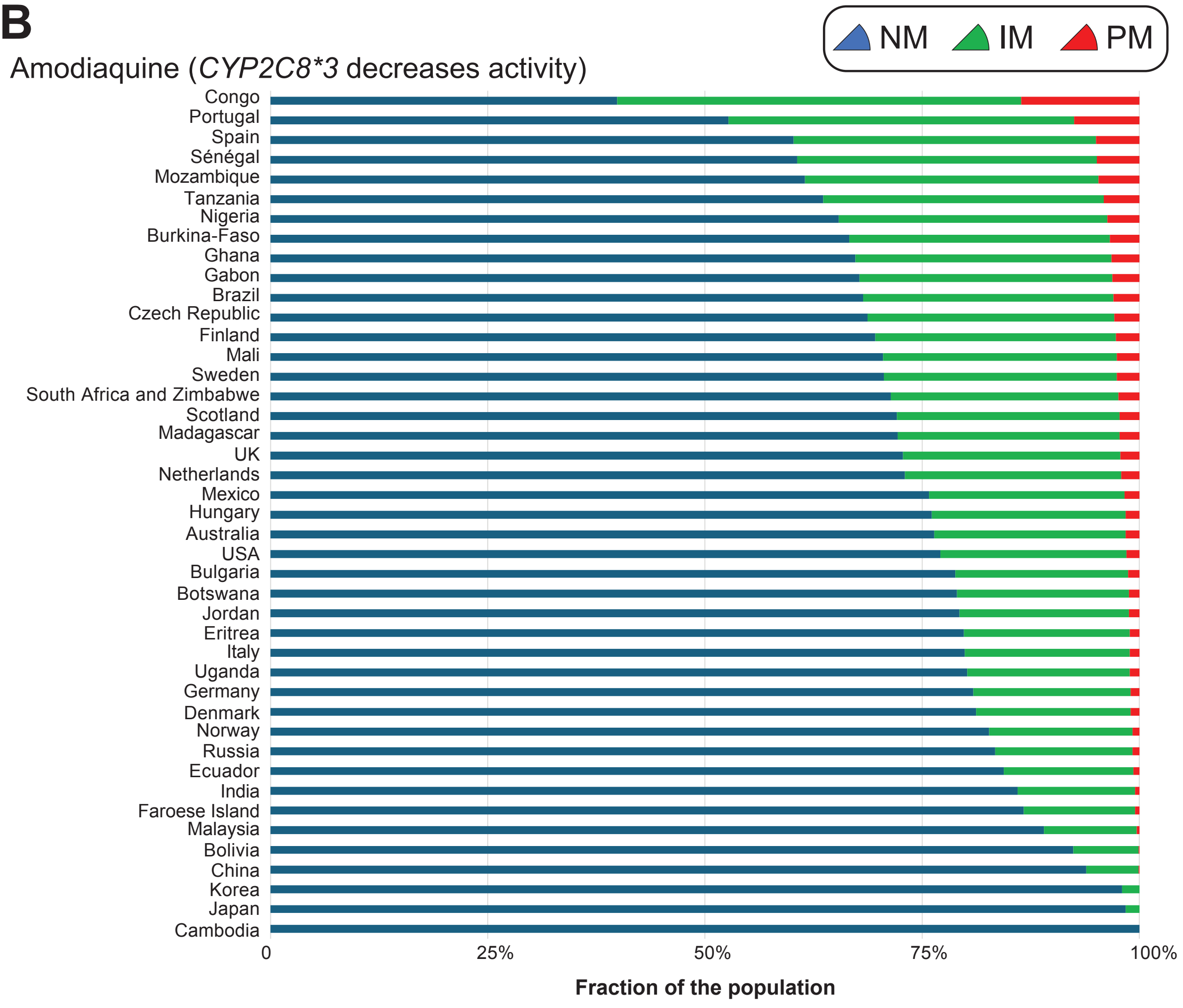

Supplement: Supplementary file 1 — Additional file 1: Fig. S1. Frequencies of intermediate and poor CYP2C8 metabolizers across analyzed countries. Countries are shown in a ranked order from high to low relative abundance of individuals with reduced CYP2C8 metabolism. IMs and PMs were defined as individuals carrying one or two reduced function alleles, respectively. A CYP2C8*3 is considered as a normal function allele. B CYP2C8*3 is considered as a decreased function allele. [file 40246_2024_610_MOESM1_ESM.pdf]
